# Editorial: Neuroimaging in dementias

**DOI:** 10.1590/1980-57642015DN94000315

**Published:** 2015

**Authors:** Claudia da Costa Leite, Carlos Alberto Buchpiguel, Marcio Luiz Figueredo Balthazar

**Affiliations:** Guests Editors

In the last few decades, neuroimaging has become one of the most relevant research areas in the field of Dementia.

Advances in different neuroimaging modalities such as structural and functional Magnetic Resonance Imaging (MRI) and Positron Emission Tomography (PET), (including FDG-PET and recent amyloid/tau tracers) have changed the role of neuroimaging in the diagnosis of dementias. Instead of only excluding other secondary diagnoses, neuroimaging has become a potential biomarker that evaluates the pathophysiology of the diseases, by means of patterns of early atrophy, metabolites, hypoperfusion, hypometabolism, structural and functional disconnectivity, vascular lesions and pathologic hallmarks of Alzheimer's disease such as amyloid and tau proteins.

This issue of Dementia & Neuropsychologia is dedicated to neuroimaging studies and reviews of dementias from different parts of Brazil and abroad. There are seven reviews, four original papers, two case reports and a new section: Neuroimaging through clinical cases. The reinforcement of Neuroimaging in different centers throughout Brazil is essential for the establishment of multicentre studies like the Alzheimer's Disease Neuroimaging Initiative (ADNI Brazil).

**Promteangtrong et al.** put together two complete and systematic reviews about structural and functional imaging in the diagnosis of dementia, with special emphasis on the diagnosis of Alzheimer's disease.

**Engler et al.** performed an excellent review about PET and the multitracer concept in the study of neurodegenerative diseases. The authors discussed the potential clinical value of new molecular probes in PET for the evaluation of neurodegenerative disorders of the brain.

**Damasceno** reviewed the most common neuroimaging findings in normal pressure hydrocephalus (NPH), as well as the latest international consensus on NPH.

**Ramalho and Castillo** presented an overview of the clinical and imaging aspects of traumatic brain injury and its importance in the cognitive decline diagnosis.

**Rocha, Nunes and Maia Jr.** reviewed the concept of motor neuron disease and fronto-temporal dementia as a unique syndrome, focusing on the clinical, pathological, genetic and imaging findings of this entity.

**Haziot et al.** reviewed the criteria of HIV-associated neurocognitive disorders (HAND) and the most relevant neuroimaging methods and findings in HAND patients.

**Coutinho et al.** studied regional brain glucose metabolism using [18F]FDG-PET and N-acetyl aspartate/Myo-inositol ratio (Naa/mI) using magnetic resonance proton spectroscopy in Alzheimer disease (AD) patients (n=32), mild cognitive impairment (MCI) patients (n=27) and in a control group (n= 28). All the evaluations studied the posterior cingulate gyrus. The paper concluded that rBGM and Naa/mI in the PCC were positively correlated in patients with MCI and AD. [18F]FDG-PET had greater accuracy than MRS for discriminating AD patients from controls.

**Sudo et al.** identified the neuroimaging profile of Vascular Mild Cognitive Impairment (VaMCI), the impact of these aspects on cognition and the neuropsychological tests that distinguished VaMCI from other groups. They found that "complex" executive function abilities consistently distinguished VaMCI from other groups, regardless of the severity of white matter hyperintensities.

**Caous et al.** described an event-related functional MRI study in which they evaluated olfactory stimuli using different emotional valence in 43 healthy subjects. They observed increased cerebral responses within the anterior cingulate, amygdaloid nucleus, and dorsolateral prefrontal, occipital and orbitofrontal cortices in positive and negative valence conditions. The response to neutral condition was less intense and not observed in the amygdaloid complex. The most significant statistical response aroused from the stimuli clusters was observed in the negative valence. As many neurological conditions such as dementias are associated with impaired olfactory function, the authors concluded that neutral stimuli may be more sensitive to early losses.

**Prado et al.** performed a systematic review of the literature on the neuroimaging investigation of frontotemporal dementia (FTD) and amyotrophic lateral sclerosis (ALS) associated with C9ORF72 mutation. They evaluated FTD, ALS and FTD-ALS patients and found consistent involvement of frontotemporal regions, but also detected alterations in other posterior cortical structures and subcortical structures such as the thalamus.

**Valente et al.** presented two cases of Creutzfeldt-Jacob disease in which the magnetic resonance imaging findings were very helpful in the final diagnosis.

**Silva et al.** reported a patient with CADASIL, genetically confirmed by NOTCH3 mutation, and skin biopsy exhibiting granular osmiophilic material deposits in the smooth muscle cells of arterioles. The authors highlighted the importance of repeat analyses of a skin biopsy, even when initially negative, in search of the ultrastructural marker of CADASIL.

**Claudia da Costa Leite** **Carlos Alberto Buchpiguel** **Marcio Luiz Figueredo Balthazar** Guests Editor

